# Revision of the fish parasitic genus *Pleopodias* Richardson, 1910 (Isopoda, Cymothoidae), with the description of a new species and key to the genus

**DOI:** 10.3897/zookeys.667.11414

**Published:** 2017-04-10

**Authors:** Kerry A. Hadfield, Nico J. Smit

**Affiliations:** 1 Water Research Group, Unit for Environmental Sciences and Management, Potchefstroom Campus, North-West University, Private Bag X6001, Potchefstroom, 2520, South Africa

**Keywords:** External parasite, Indian Ocean, South Africa, *Pleopodias diaphus*, *Pleopodias elongatus*, *Pleopodias vigilans*, *Pleopodias nielbrucei*, *Pleopodias superatus*

## Abstract

The cymothoid genus, *Pleopodias* Richardson, 1910, is revised and a new species from South Africa is recorded. *Pleopodias nielbrucei* **sp. n.** can be distinguished by large eyes covering majority of the cephalon (almost in contact), antennula bases wide apart, antenna extending to middle of pereonite 2, subtruncate pleotelson, pereopod 7 with numerous acute robust setae on the propodus as well as the carpus, and the uropod exopod longer than the endopod. The three known species, *Pleopodias diaphus* Avdeev, 1975; *P. elongatus* Richardson, 1910; and *P. vigilans* Richardson, 1911 are also redescribed. *Pleopodias nielbrucei* **sp. n.** differs from these known species in both morphological characters as well as geographical distribution. A key to the *Pleopodias* species is provided.

## Introduction

Depending on the genus or species, cymothoids can be located on the external surfaces or inside the branchial, buccal or body cavities of their fish host (Smit et al. 2014). The genus *Pleopodias* Richardson, 1910, is a small genus of fish-parasitic isopods which has been rarely studied since it was founded. These cymothoids most likely occur on the external surfaces of their fish hosts and to date there are three recognised species. *Pleopodias diaphus* Avdeev, 1975 (*Pleopodias superatus* Williams & Williams, 1986 is the junior synonym of this species) is known only from Japan; *P. elongatus* Richardson, 1910 is known only from the Philippine Islands; and *P. vigilans* Richardson, 1911 has been reported only from the coast of Sudan.

While examining museum material housed in the Iziko South African Museum, Cape Town, a specimen collected off the coast of South Africa identified as belonging to the genus *Pleopodias* was observed. Several fish parasitic cymothoid genera from southern Africa have recently been revised (Hadfield et al. 2010, 2013, 2014, 2015), but this is the first record of *Pleopodias* from South Africa.

The *Pleopodias* species from South Africa differs from the three known species in both morphological characteristics as well as geographical distribution, Discovery of this specimen provided the opportunity to revise the genus, add a new species, and provide a key to all species of *Pleopodias*.

## Methods

*Pleopodias* type material was borrowed or drawn at the respective museums. The *Pleopodias* specimen loaned from the Iziko South African Museum was collected from a RV Africana Cruise in 1988, off the coast of South Africa. Type material was not dissected and all isopods were processed according to the techniques in Hadfield et al. (2010, 2013). The cymothoid species descriptions were prepared in DELTA (DEscriptive Language for TAxonomy) format using a general Cymothoidae character set used previously (see Hadfield et al. 2016). Isopod classification follows that of Brandt and Poore (2003) and host nomenclature was sourced and verified from FishBase (Froese and Pauly 2017) and Catalog of Fishes (Eschmeyer 2017).

*Abbreviations*. MNHN—Muséum national d’Histoire naturelle, Paris; SAMC—South African Museum, Cape Town; USNM—National Museum of Natural History, Smithsonian Institution, Washington; TL—total length; W—width.

## Taxonomy

### Suborder Cymothoida Wägele, 1989

#### Superfamily Cymothooidea Leach, 1814

##### Family Cymothoidae Leach, 1814

###### Genus Pleopodias

Taxon classificationAnimaliaIsopodaCymothoidae

Richardson, 1910

Pleopodias Richardson, 1910: 25–26.—Barnard, 1936: 166–167.—Bruce, 1987: 87.—Trilles, 1994: 109.

####### Type species.

*Pleopodias elongatus* Richardson, 1910, by monotypy.

####### Diagnosis.

Body elongate; cephalon slightly immersed in pereonite 1, posterior margin not trilobed; eyes large and distinct. Rostrum folded back, lying between antennula bases, not concealing basal articles. Antennula long and narrow, extending past cephalon posterior margins; antenna longer than antennula, extending to or beyond pereonite 2, articles 3–6 elongate. Pereon most narrow at pereonite 1; pleon narrower than pereon with pleonites progressively getting narrower from pleonite 1 to 5; pleotelson elongate, 1.2–1.8 times longer than wide. Pereonite 2 shortest in length, pereonite 6 longest. Uropods long and narrow, extending beyond the posterior margin of the pleotelson. Pereopod 7 longer than other pereopods with the merus, carpus and propodus on pereonite 7 elongated.

####### Remarks.

*Pleopodias* can be identified by the long antennae, with the antennula shorter than the antenna; body most narrow at pereonite 1; narrow pleon with the width of the pleonites decreasing from pereonite 1 to 5; pereopod 7 longer and more elongate than other pereopods; uropods extending past the posterior margin of the pleotelson; and a longer than wide pleotelson.

The original diagnosis for this genus was provided by Richardson (1910) but was based on the only species known at that time, *P. elongatus*. In his report on a *Pleopodias* sp. from the Andaman Islands, Barnard (1936) pointed out that of some of the generic characters used by Richardson to define the genus (i.e. antennula articles 2 and 3 expanded, and pleopods visible in dorsal view) were shared with *Anilocra* Leach, 1818 and thus not very informative. Bruce (1987) revised both *Pleopodias* and *Anilocra* simultaneously and was able to provide a number of differences between the two genera. According to Bruce (1987), *Pleopodias* has a narrow pleon, getting strongly narrower towards the posterior (not always narrower in *Anilocra*); antennula articles 4–8 are elongate (not in *Anilocra*); large robust setae on the maxilla and the medial lobe is distinct (acute, simple robust setae and the maxilla is partially fused in *Anilocra*); article 3 of the mandible palp is slim and longer than article 2 (article is stout and short in *Anilocra*); and pereopod 7 with more robust setae than observed in *Anilocra*. Bruce (1987) also included mouthpart and pleopod morphology in the generic diagnosis of *Pleopodias*, however, as not all species have these characters noted, we have refrained from adding them into the currently revised diagnosis.

####### Key to the species of the genus *Pleopodias*

This key is based on the morphological characters of the gravid female:

**Table:** 

1	Uropod rami the same length; antennula bases close together or in contact	**2**
–	Uropod exopod longer than endopod; antennula bases widely separated	**3**
2	Antennula bases contiguous; antenna extending to middle of pereonite 3; pleotelson posterior margin deeply emarginate; eye size a third of cephalon width	***P. diaphus***
–	Antennula bases narrowly separated; antenna extending to posterior of pereonite 2; pleotelson posterior margin rounded; eye size a quarter of cephalon width	***P. elongatus***
3	Antenna extending to posterior of pereonite 2; pleotelson posterior margin rounded, with caudomedial point; eye 0.4 times width of cephalon; pereopod 7 without robust setae	***P. vigilans***
–	Antenna extending to middle of pereonite 2; pleotelson posterior margin subtruncate; eye 0.5 times width of cephalon; pereopod 7 with acute robust setae on propodus and carpus	***P. nielbrucei* sp. n.**

###### Pleopodias diaphus

Taxon classificationAnimaliaIsopodaCymothoidae

Avdeev, 1975

Pleopodias diaphus Avdeev, 1975: 254–256, figs 1–11.—Bruce & Harrison-Nelson, 1988: 600.—Trilles, 1994: 109.—Yamauchi, 2009: 477–479, figs 7–8.
Pleopodias superatus Williams & Williams, 1986: 656, figs 62–68.

####### Material examined.

Female holotype of *Pleopodias superatus* (26.5 mm TL, 10.4 mm W), caught in a shrimp net off Honshu Island, Japan, 11 April 1969 (USNM 231069).

*Holotype.* Non-ovigerous female, collected from the East-China Sea (Sea of Japan), from the body of *Diaphus coeruleus* (TINRO AGK 74190). *Paratypes*. Non-ovigerous females, same information as holotype (TINRO APK 74191–74195). Not examined.

**Figure 1.**
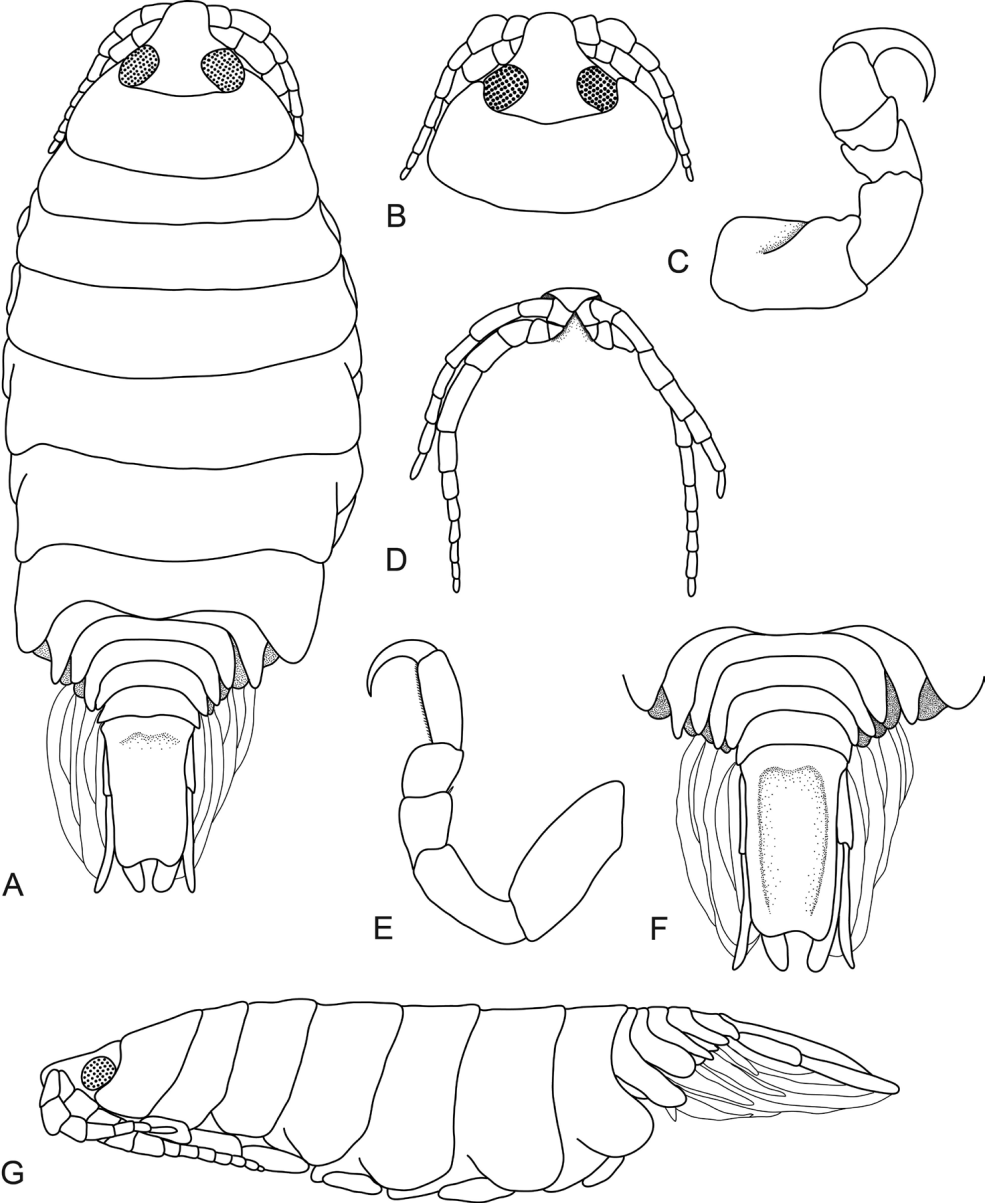
*Pleopodias diaphus* Avdeev, 1975 (USNM 231069), female (26 mm), (originally designated as the holotype of *Pleopodias superatus* Williams & Williams, 1986). **A** dorsal view **B** dorsal view of pereonite 1 and cephalon **C** pereopod 1 **D** ventral view of cephalon **E** pereopod 7 **F** dorsal view of pleon and pleotelson **G** lateral view.

####### Description.

Female holotype of *Pleopodias superatus*. Length 26.5 mm, width 10.4 mm.

*Body* oval, 2.4 times as long as greatest width, dorsal surfaces smooth and polished in appearance, widest at pereonite 5, most narrow at pereonite 1, lateral margins subparallel. *Cephalon* 0.8 times longer than wide, visible from dorsal view, subtriangular. *Frontal margin* truncate, thickened and ventrally folded. *Eyes* oval with distinct margins; one eye 0.3 times width of cephalon, 0.4 times length of cephalon. *Pereonite 1* smooth, anterior border slightly indented, anterolateral angle narrowly rounded, extending to middle of the eye. Posterior margins of pereonites 1–4 smooth and straight, 5–7 slightly curved laterally, posterior margin of pereonite 7 produced medially. Coxae 2–3 narrow, with posteroventral angles rounded; 4–7 small and narrow, not extending past pereonite margin. Pereonites 1–5 increasing in length and width; 6–7 decreasing in length and width. Pereonite 7 partially overlapping pleonite 1. *Pleonites* posterior margin smooth, mostly concave. Pleonite 1 widest, visible in dorsal view. Pleonite 2 not overlapped by pereonite 7; posterolateral angles of pleonite 2 narrowly rounded. Pleonites 3–5 progressively getting smaller; pleonite 5 not overlapped by lateral margins of pleonite 4, posterior margin slightly concave. *Pleotelson* 1.8 times as long as anterior width, dorsal surface slightly depressed, lateral margins straight, posterior margin with medial notch.

*Antennula* thinner and shorter than antenna, contiguous bases, consisting of 8 articles; peduncle articles 1 and 2 distinct and articulated; articles 2–3 expanded; extending to pereonite 2. *Antenna* consisting of 12 articles; extending to middle of pereonite 3. *Pereopod 1* basis 1.6 times as long as greatest width; ischium 0.7 times as long as basis; merus proximal margin without bulbous protrusion; carpus with straight proximal margin; propodus 1.2 times as long as wide; dactylus slender, 1.5 times as long as propodus, 2.4 times as long as basal width. *Pereopod 7* longer than other pereopods, basis 2.2 times as long as greatest width; ischium 0.8 times as long as basis, without protrusions; merus proximal margin without bulbous protrusion, 1.2 times as long as wide, 0.5 times as long as ischium; carpus 0.9 times as long as wide, 0.4 times as long as ischium, without bulbous protrusion; propodus with numerous acute robust setae, 2.6 times as long as wide, 0.8 times as long as ischium; dactylus slender, 0.8 times as long as propodus, 3.3 times as long as basal width. *Uropod* longer than the pleotelson, rami subequal. *Endopod* apically rounded. *Exopod* apically narrowly rounded.

####### Distribution.

Sea of Japan (Avdeev 1975) and off Honshu, Japan (Williams and Williams 1986; Yamauchi 2009).

####### Hosts.

Anterior to the dorsal fin of *Diaphus coeruleus* (Blue lantern fish) (Avdeev 1975).

####### Remarks.

*Pleopodias diaphus* has an ovate shape, contiguous antennula bases, and an emarginated pleotelson posterior margin. The uropod rami are approximately the same length and the eyes are large (each eye approximately a third of the cephalon width).

It was originally described from Japan, with several drawings and a brief description in Russian. In 1986, Williams and Williams described a new species, *Pleopodias superatus* which shared many similarities with *P. diaphus*. After comparisons of the notched pleotelson as well as the antennae and somatic morphology, Bruce and Harrison-Nelson (1988) synonymised it with *P. diaphus*. Despite numerous attempts, the type specimens of *P. diaphus* could not be obtained for inclusion in the present study; however, the types do exist and the species is eminently recognisable from the original illustrations and is therefore not a *nomen dubium* or *species inquirenda*. Avdeev (1975) reported the types as immature females but both the size and drawings indicate they are still adult females (non-ovigerous) and therefore suitable for a valid species description. Both species (*P. diaphus* and *P. superatus*) are well illustrated, readily recognised, from the same region, and appear identical, thus the synonymy of the two species by Bruce and Harrison-Nelson (1988) is here upheld until a detailed redescription of the original type material of *P. diaphus* indicates otherwise.

As the type material for *P. diaphus* could not be obtained the redescription provided here is based on the holotype of *P. superatus* housed at USNM. This redescription includes updated measurements and characteristics which are comparable to the other *Pleopodias* species in this paper. This modern description of the type material of *P. superatus* will also aid future research into its current status as junior synonym of *P. diaphus*.

Pleopods and mouthparts of specimens identified as *P. diaphus* were drawn and described by Yamauchi (2009). The specimens Yamauchi (2009) examined largely conformed to the above description; however, the body size differed with the three more recent samples (12–27 mm in length) being more slender (3.4–3.9 times as long as wide).

###### Pleopodias elongatus

Taxon classificationAnimaliaIsopodaCymothoidae

Richardson, 1910

Pleopodias elongatus Richardson, 1910: 26–27, fig. 25.—Nierstrasz, 1931: 133.—Avdeev, 1975: 89, fig. 3.—Bruce, 1987: 89, fig. 3.

####### Material examined.

*Holotype*. Ovigerous female (20 mm), off Matocot Point, Philippine Islands, 8 June 1908, 170 fathoms (= 311 m depth), station 5268, coll. U .S. Bureau of Fisheries *Albatross* Philippine Expedition 1907-08 (USNM 40917). Also noted: bottom third of the pleotelson folded in.

**Figure 2.**
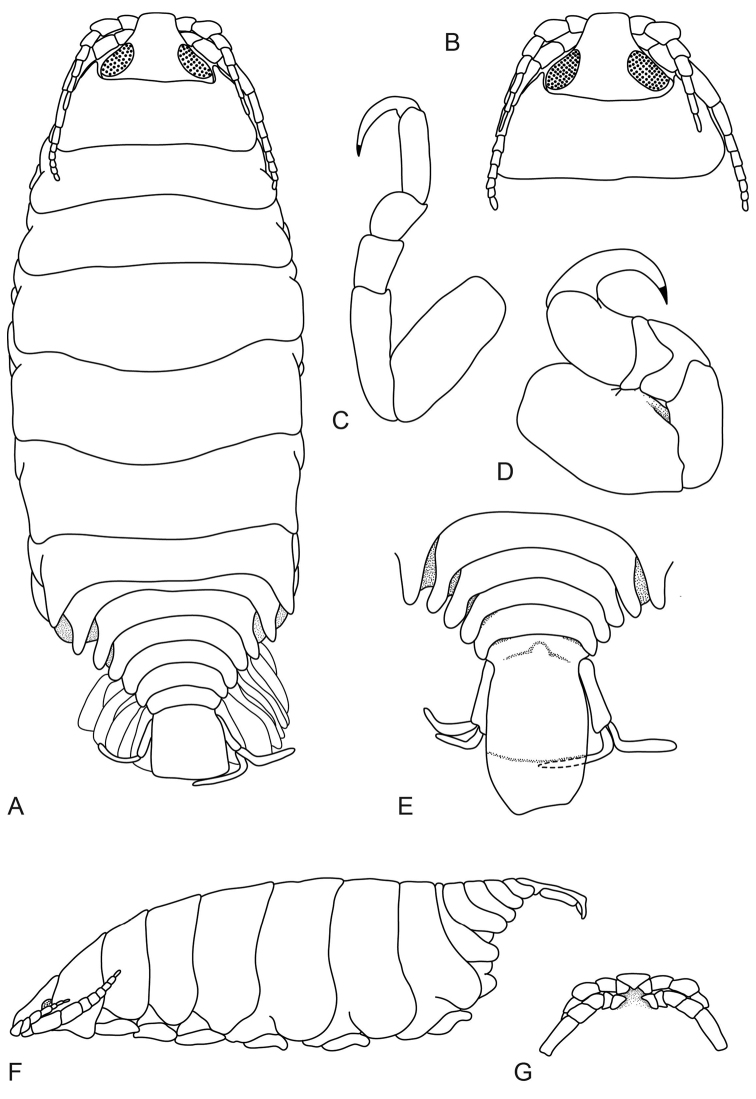
*Pleopodias elongatus* Richardson, 1910 (USNM 40917), female holotype (20 mm). **A** dorsal view **B** dorsal view of pereonite 1 and cephalon **C** pereonite 7 **D** pereopod 1 **E** dorsal view of pleon and pleotelson **F** lateral view **G** ventral view of cephalon.

####### Description.

*Female holotype.* Length 20 mm, width 7.5 mm.

*Body* elongate, 2.7 times as long as greatest width, dorsal surfaces smooth and polished in appearance, widest at pereonite 4 and pereonite 5, most narrow at pereonite 1, lateral margins subparallel. *Cephalon* 0.7 times longer than wide, visible from dorsal view, subtriangular. *Frontal margin* thickened, ventrally folded and truncate. *Eyes* oval with distinct margins, one eye 0.25 times width of cephalon; 0.4 times length of cephalon. *Pereonite 1* smooth, anterior border straight, anterolateral angle acute, anteriorly produced, extending to one third of the eye. Posterior margins of pereonites smooth and slightly curved laterally. Coxae 2–4 narrow, with posteroventral angles rounded; 5–7 small and narrow, not extending past pereonite margin, with posteroventral angles curved. Pereonites 1–4 increasing in length and width; 5–7 decreasing in length and width. *Pleonites* posterior margin smooth, mostly concave. Pleonite 1 widest, visible in dorsal view. Pleonite 1 and 2 not overlapped by pereonite 7; posterolateral angles of pleonite 2 rounded. Pleonites 3–5 progressively getting smaller; pleonite 5 not overlapped by lateral margins of pleonite 4, posterior margin straight. *Pleotelson* 1.6 times as long as anterior width, dorsal surface slightly depressed, lateral margins weakly convex, posterior margin rounded and damaged.

*Antennula* thinner than antenna, length longer than antenna, bases narrowly separated, consisting of 8 articles; peduncle articles 1 and 2 distinct and articulated; articles 2–3 expanded; extending to middle of pereonite 1. *Antenna* consisting of 11 articles; extending to middle of pereonite 2. *Pereopod 1* basis 1.6 times as long as greatest width; ischium 0.7 times as long as basis; merus proximal margin without bulbous protrusion; carpus with straight proximal margin; propodus 1.4 times as long as wide; dactylus moderately slender, 1.3 times as long as propodus, 2.4 times as long as basal width. *Pereopod 7* longer than other pereopods, basis 2.3 times as long as greatest width; ischium 0.8 times as long as basis, without protrusions; merus proximal margin without bulbous protrusion, 1.3 times as long as wide, 0.4 times as long as ischium; carpus 1.3 times as long as wide, 0.4 times as long as ischium, with slight bulbous protrusion; propodus 3.3 times as long as wide, 0.8 times as long as ischium; dactylus slender, 0.9 times as long as propodus, 3.7 times as long as basal width. *Uropod* longer than the pleotelson, rami subequal. *Endopod* apically slightly pointed. *Exopod* apically narrowly rounded.

####### Hosts.

Not known.

####### Distribution.

Philippine Islands (Richardson 1910; Bruce 1987).

####### Remarks.

*Pleopodias elongatus* can be distinguished by the small eyes (each eye a quarter of the cephalon width), antennula bases narrowly separated, antenna extending to the middle of pereonite 2, rounded pleotelson, and uropod rami approximately the same length.

The only other species recorded from the Pacific is *P. diaphus* (from Japan). *Pleopodias elongatus* differs from *P. diaphus* in having antennula bases narrowly separated (*P. diaphus* bases in contact), shorter antenna (*P. diaphus* antenna extend to posterior of pereonite 3), absence of robust setae on propodus of pereopod 7 (*P. diaphus* has numerous acute robust setae), a rounded pleotelson (*P. diaphus* pleotelson subquadrate with a deeply emarginated medial notch), and smaller eyes (*P. diaphus* eyes cover a third of the cephalon width).

###### Pleopodias vigilans

Taxon classificationAnimaliaIsopodaCymothoidae

Richardson, 1911

Pleopodias vigilans Richardson, 1911: 525–526.

####### Material examined.

*Holotype.* Female (28 mm TL, 11 mm W), 9 July 1883, collected from the *Talisman*, St. DR71 (dredge), 640 m depth, coast of Sudan, MNHN-IU-2014-12188 (= MNHN-Is2460). Also noted: the specimen is black with the pleotelson folded in.

**Figure 3.**
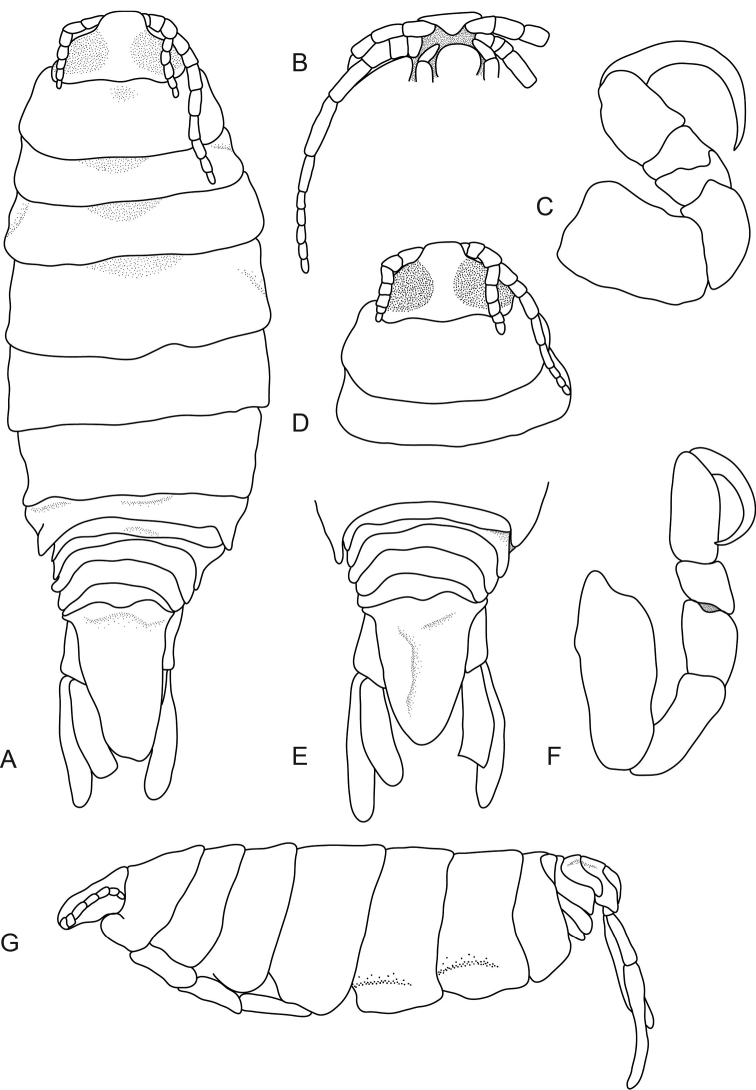
*Pleopodias vigilans* Richardson, 1911 (MNHN-IU-2014-12188), female holotype (28 mm). **A** dorsal view **B** ventral view of cephalon **C** pereopod 1 **D** dorsal view of pereonite 1, pereonite 2 and cephalon **E** dorsal view of pleon and pleotelson **F** pereopod 7 **G** lateral view.

####### Description.

*Female holotype.* Length 28 mm, width 11 mm.

*Body* elongate, 2.9 times as long as greatest width, dorsal surfaces slightly bumpy, widest at pereonite 4, most narrow at pereonite 1. *Cephalon* 0.6 times longer than wide, visible from dorsal view, subtriangular. *Frontal margin* thickened, ventrally folded and truncate. *Eyes* not clearly defined; one eye 0.4 times width of cephalon, 0.6 times length of cephalon. *Pereonite 1* smooth, anterior border slightly irregular, anterolateral angle narrowly rounded. Posterior margins of pereonites smooth and straight. Coxae 2–3 wide, with posteroventral angles rounded; 4–7 small and narrow, extending past pereonite margin. Pereonites 1–4 increasing in length and width; 5–7 decreasing in length and width. *Pleonites* posterior margin not smooth, mostly concave. Pleonite 1 widest, slightly visible in dorsal view. Pleonite 2 not overlapped by pereonite 7; posterolateral angles of pleonite 2 rounded. Pleonites 3–5 progressively getting smaller; pleonite 5 with posterolateral angles rounded, posterior margin produced medially. *Pleotelson* 1.2 times as long as anterior width, dorsal surface slightly depressed, lateral margins posteriorly narrow, posterior margin converging to rounded caudomedial point.

*Antennula* thinner and shorter than antenna, bases widely separated, consisting of 8 articles; peduncle articles 1 and 2 distinct and articulated; extending past the posterior margin of cephalon. *Antenna* consisting of 11 articles, extending to posterior of pereonite 2. *Pereopod 1* basis 1.6 times as long as greatest width; ischium 0.7 times as long as basis; merus proximal margin without bulbous protrusion; carpus with straight proximal margin; propodus 1.5 times as long as wide; dactylus slender, 1.8 times as long as propodus. *Pereopod 7* longer than other pereopods, basis 2.4 times as long as greatest width; ischium 0.6 times as long as basis, without protrusions; merus proximal margin without bulbous protrusion, 1.3 times as long as wide, 0.6 times as long as ischium; carpus 1.1 times as long as wide, 0.4 times as long as ischium, without bulbous protrusion; propodus 2.3 times as long as wide, 0.9 times as long as ischium; dactylus slender, 1.1 times as long as propodus, 3.7 times as long as basal width. *Uropod* longer than the pleotelson; peduncle 0.4 times longer than rami, peduncle lateral margin without setae, apices broadly rounded. *Endopod* apically rounded, 3.5 times as long as greatest width. *Exopod* extending beyond posterior of endopod, 5 times as long as greatest width, apically rounded.

####### Distribution.

Sudan (Richardson 1911).

####### Hosts.

Not known.

####### Remarks.

*Pleopodias vigilans* can be identified by the antennula bases being widely separated, large eyes occupying majority of the cephalon, antenna extending to posterior of pereonite 2, uropodal exopod longer than endopod, and rounded pleotelson with a caudomedial point.

Only one specimen of this species has ever been collected. Due to the age and condition of the specimen, it was not possible to see some of the characters usually associated with *Pleopodias* (i.e. robust setae on pereopod 7 etc.).

No figures of the specimen were provided in the original description, and as no other collections have been made since, no drawings of the specimen have ever been produced. This redescription provides the first illustrated figures of *P. vigilans* and will help future identifications of this species. Fresh collections of this species could prove valuable in adding to this information, along with information on the mouthparts and pleopods of this species.

###### Pleopodias nielbrucei sp. n.

Taxon classificationAnimaliaIsopodaCymothoidae

http://zoobank.org/4063D2C2-A939-4E9C-8F1E-F759246443DD

####### Material examined.

*Holotype.* Female (30mm TL; 9mm W), RV Africana Cruise 060 (34°46.6'S 18°02.5'E), Station A7033-060-14-03M, South Africa, 14 March 1988, 702m depth (SAMC A088881). *Paratype*. Male (20mm TL; 5 mm W), same info as holotype (SAMC A43478).

**Figure 4.**
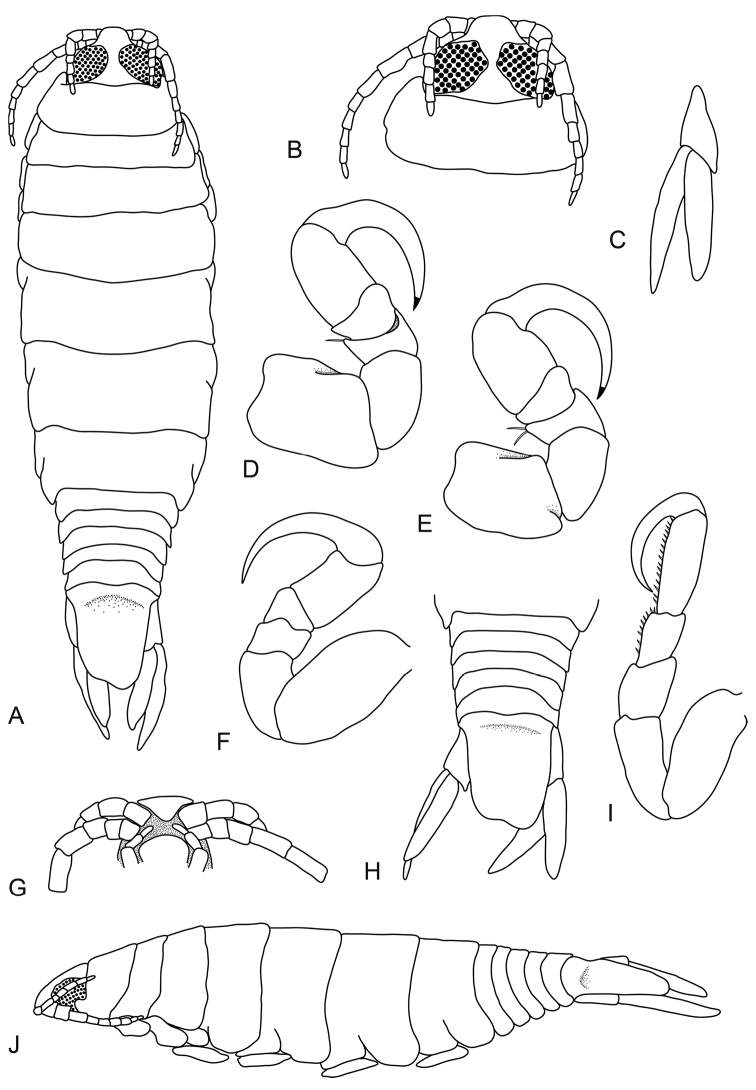
*Pleopodias nielbrucei* sp. n. (SAMC A088881), female holotype (30 mm). **A** dorsal view **B** dorsal view of pereonite 1 and cephalon **C** uropod **D** pereopod 1 **E** pereopod 2 **F** pereopod 6 **G** ventral view of cephalon **H** dorsal view of pleon and pleotelson **I** pereopod 7 **J** lateral view.

**Figure 5.**
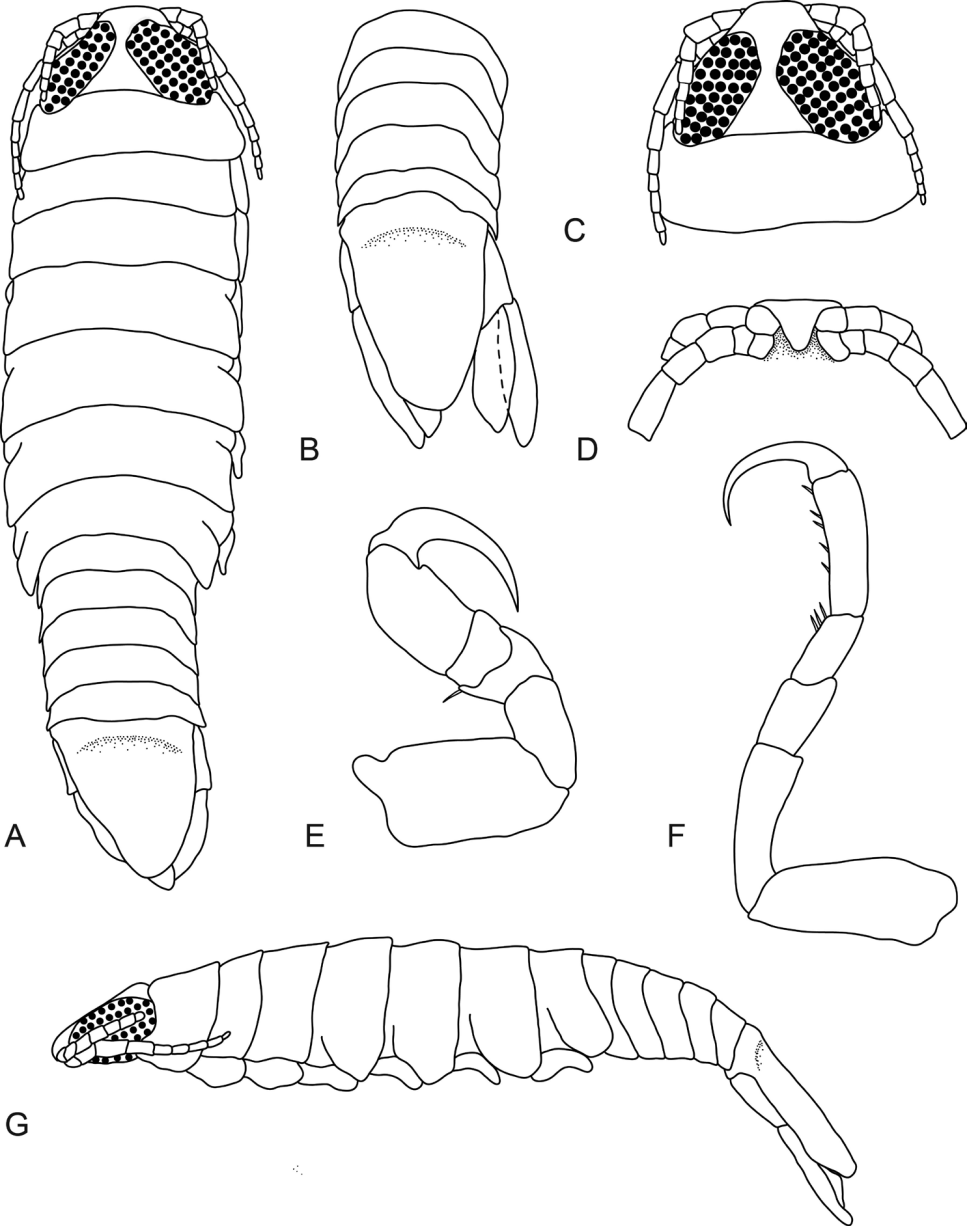
*Pleopodias nielbrucei* sp. n. (SAMC A43478), male paratype (20 mm). **A** dorsal view **B** dorsal view of pleon and pleotelson **C** dorsal view of pereonite 1 and cephalon **D** ventral view of cephalon **E** pereopod 1 **F** pereopod 7 **G** lateral view.

####### Description.

*Female holotype.* Length 30 mm, width 9 mm.

*Body* narrow, 3.3 times as long as greatest width, dorsal surfaces smooth and polished in appearance, widest at pereonite 5, most narrow at pereonite 1, lateral margins subparallel. *Cephalon* 0.7 times longer than wide, visible from dorsal view, subtriangular. *Frontal margin* thickened, ventrally folded and truncate. *Eyes* oval with distinct margins, one eye almost 0.5 times width of cephalon; 0.6 times length of cephalon. *Pereonite 1* smooth, anterior border slightly indented, anterolateral angle narrowly rounded, extend to one third of the eye. Posterior margins of pereonites smooth and slightly curved laterally. Coxae 2–3 wide, with posteroventral angles rounded; 4–7 small and narrow, not extending past pereonite margin. Pereonites 1–5 increasing in length and width; 6–7 decreasing in length and width. *Pleonites* posterior margin smooth, mostly concave. Pleonite 1 widest, visible in dorsal view. Pleonite 2 not overlapped by pereonite 7; posterolateral angles of pleonite 2 narrowly rounded. Pleonites 3–5 similar in form to pleonite 2; pleonite 5 not overlapped by lateral margins of pleonite 4, posterior margin produced medially. *Pleotelson* 1.2 times as long as anterior width, dorsal surface slightly depressed, lateral margins straight, posterior margin subtruncate.

*Antennula* thinner and shorter than antenna, bases widely separated, consisting of 8 articles; peduncle articles 1 and 2 distinct and articulated; extending to anterior of pereonite 1. *Antenna* consisting of 11 articles, extending to middle of pereonite 2. *Pereopod 1* basis 1.4 times as long as greatest width; ischium 0.7 times as long as basis; merus with simple setae, proximal margin without bulbous protrusion; carpus with rounded proximal margin; propodus 1.5 times as long as wide; dactylus moderately slender, 1.7 times as long as propodus, 3.3 times as long as basal width. *Pereopod 2* propodus 1.4 times as long as wide; merus with simple setae; dactylus 1.7 times as long as propodus. *Pereopod 6* basis twice as long as greatest width, ischium 0.6 times as long as basis, propodus 1.7 times as long as wide, dactylus 1.8 times as long as propodus. *Pereopod 7* longer than other pereopods, basis 2.3 times as long as greatest width; ischium 0.8 times as long as basis, without protrusions; merus proximal margin without bulbous protrusion, 1.2 times as long as wide, 0.6 times as long as ischium; carpus with numerous acute robust setae, 1.4 times as long as wide, 0.5 times as long as ischium, without bulbous protrusion; propodus with numerous acute robust setae, 2.8 times as long as wide, as long as ischium; dactylus slender, as long as propodus, 3.5 times as long as basal width. *Uropod* longer than the pleotelson, peduncle 0.4 times longer than rami, peduncle lateral margin without setae. *Endopod* apically slightly pointed, 5 times as long as greatest width, terminating without setae. *Exopod* extending beyond posterior of endopod, apically narrowly rounded, terminating without setae.

*Male paratype.* Length 20 mm, width 5 mm.

Male similar to female but smaller. Body rectangular, body 3.5 times as long as wide. *Antennula* bases separated, consisting of 8 articles, extending to posterior margin of cephalon. *Antenna* consisting of 12 articles, extending to middle of pereonite 2. Eyes slightly separated, one eye almost 0.5 times width of cephalon; 0.7 times length of cephalon.

####### Etymology.

Named in honour of Dr Niel Bruce, in recognition of his significant contribution to the taxonomy of isopods, specifically that of fish parasitic cymothoids.

####### Distribution.

Off the coast of Cape Town, South Africa.

####### Hosts.

Not known.

####### Remarks.

*Pleopodias nielbrucei* sp. n. can be identified by the narrow body, large eyes covering majority of the cephalon (almost in contact), antennula bases wide apart, antenna extending to middle of pereonite 2, subtruncate pleotelson, pereopod 7 with numerous acute robust setae on the propodus as well as the carpus, and the uropodal exopod longer than the endopod.

This is the first named *Pleopodias* species from the southern hemisphere (not including the unknown *Pleopodias* sp. mentioned below). It differs from the other three known species in having larger eyes and a more elongate body, as well as a shorter and more quadrate pleotelson and antennula bases which are further apart than the other species. *Pleopodias nielbrucei* sp. n. also has a less graduated pleon (the pleonites do not decrease in width from pleonite 1 to 5 as prominently as *P. diaphus* and *P. elongatus*).

###### Pleopodias

Taxon classificationAnimaliaIsopodaCymothoidae

sp.

Pleopodias elongatus Barnard, 1936: 167–168, fig. 7f–g. (not P. elongatus Richardson, 1910).
Pleopodias sp. Bruce, 1987: 87, figs 1–2.—Trilles, 1994: 109.

####### Material.

Ovigerous female (14.5 mm TL), 232 km north of Port Hedland, Western Australia, 10 Oct 1982, 298-300 m depth, coll: L. Marsh & S. Slack-Smith on FRV Soela (WAM 607-80). Also noted: Specimen is crushed within the tube. Not examined.

Ovigerous female (15.5 mm TL), 370–419 fathoms, north of Andaman Islands (14°13'N; 93°40'E). Not examined.

####### Distribution.

Andaman Islands and Australia (Barnard 1936; Bruce 1987).

####### Hosts.

Not known.

####### Remarks.

Bruce (1987) reported what appears to be an undescribed *Pleopodias* species; however, the Australian specimen is crushed and the whereabouts of Barnard’s specimen is unknown. This species differs from *P. elongatus* (which it was originally identified as by Barnard) in having a sub-truncate and very narrow pleotelson, uropods which extend to the posterior margin of the pleotelson, antennula bases contiguous, a shorter rostrum, larger eyes, and a less laterally rounded pereonite 7. A tentative description of the Australian specimen was provided by Bruce (1987) but more specimens (in good condition) are required in order to describe the species.

## Supplementary Material

XML Treatment for Pleopodias

XML Treatment for Pleopodias diaphus

XML Treatment for Pleopodias elongatus

XML Treatment for Pleopodias vigilans

XML Treatment for Pleopodias nielbrucei

XML Treatment for Pleopodias
